# Resistance, rebound, and recurrence regrowth patterns in pediatric low-grade glioma treated by MAPK inhibition: A modified Delphi approach to build international consensus-based definitions—International Pediatric Low-Grade Glioma Coalition

**DOI:** 10.1093/neuonc/noae074

**Published:** 2024-05-14

**Authors:** Patricia O’Hare, Tabitha Cooney, Peter de Blank, David H Gutmann, Mark Kieran, Till Milde, Jason Fangusaro, Michael Fisher, Shivaram Avula, Roger Packer, Kohei Fukuoka, Kshitij Mankad, Sabine Mueller, Angela J Waanders, Enrico Opocher, Eric Bouffet, Eric Raabe, Natacha Entz Werle, Amedeo A Azizi, Nathan J Robison, Pablo Hernáiz Driever, Mark Russo, Netteke Schouten, Cornelis M van Tilburg, Astrid Sehested, Jacques Grill, Pratiti Bandopadhayay, John-Paul Kilday, Olaf Witt, David M Ashley, Birgit Betina Ertl-Wagner, Uri Tabori, Darren R Hargrave

**Affiliations:** Department of Paediatric Oncology, Royal Belfast Hospital for Sick Children, Northern Ireland, UK; Dana-Farber/Boston Children’s Cancer and Blood Disorders Center, Boston, Massachusetts, USA; Department of Pediatrics, Harvard Medical School, Broad Institute, Cambridge, Massachusetts, USA; Day One Biopharmaceuticals, Boston, Massachusetts, USA; Dana-Farber/Boston Children’s Cancer and Blood Disorders Center, Boston, Massachusetts, USA; Department of Pediatrics, Harvard Medical School, Broad Institute, Cambridge, Massachusetts, USA; University of Cincinnati College of Medicine and Cincinnati Children’s Hospital Medical Center, Cincinnati, Ohio, USA; Department of Neurology, Washington University School of Medicine, St. Louis, Missouri, USA; Day One Biopharmaceuticals, Boston, Massachusetts, USA; Clinical Pediatric Oncology, Hopp Children’s Cancer Center (KiTZ), Heidelberg, Germany; Heidelberg University Hospital, Heidelberg, Germany; German Cancer Research Center, National Center for Tumor Diseases (NCT), Heidelberg, Germany; Aflac Cancer and Blood Disorders Center, Children’s Healthcare of Atlanta and Emory University School of Medicine, Atlanta, Georgia, USA; Division of Oncology, The Children’s Hospital of Philadelphia, Philadelphia, Pennsylvania, USA; Department of Radiology, Alder Hey Children’s NHS Foundation Trust, Liverpool, UK; Brain Tumor Institute, Center for Neuroscience and Behavioral Medicine, Children’s National Hospital, Washington, District of Columbia, USA; Department of Hematology/Oncology, Saitama Children’s Medical Center, Saitama, Japan; Department of Radiology, Great Ormond Street Hospital for Children NHS Foundation Trust, Department of Radiology, London, UK; Department of Neurology, Neurosurgery and Pediatrics, University of California, San Francisco, San Francisco, California, USA; Department of Pediatrics, Northwestern University Feinberg School of Medicine, Chicago, Illinois, USA; Paediatric Haematology, Oncology and Stem Cell Transplant Division, Padua University Hospital, Padua, Italy; The Hospital for Sick Children and Department of Paediatrics, University of Toronto, Toronto, Ontario, Canada; Division of Pediatric Oncology, Department of Oncology, Johns Hopkins University School of Medicine, Baltimore, Maryland, USA; Department of Pathology, Johns Hopkins University School of Medicine, Baltimore, Maryland, USA; Pediatric Onco-Hematology Department, University Hospital of Strasbourg. UMR CNRS 7021, University of Strasbourg, Strasbourg, France; Department of Pediatrics and Adolescent Medicine and Comprehensive Centre of Pediatrics, Medical University of Vienna, Vienna, Austria; Division of Hematology & Oncology, Children’s Hospital Los Angeles, University of Southern California Keck School of Medicine, Los Angeles, California, USA; Charité-Universitätsmedizin Berlin, corporate member of Freie Universität Berlin and Humboldt-Universität zu Berlin, German HIT-LOGGIC-Registry for LGG in children and adolescents, Department of Pediatric Oncology/Hematology, Berlin, Germany; Novartis Pharmaceuticals Corporation, East Hanover, New Jersey, USA; Princess Maxima Center for Pediatric Oncology, Utrecht, Netherlands; Clinical Pediatric Oncology, Hopp Children’s Cancer Center (KiTZ), Heidelberg, Germany; Heidelberg University Hospital, Heidelberg, Germany; German Cancer Research Center, National Center for Tumor Diseases (NCT), Heidelberg, Germany; Department of Paediatrics and Adolescent Medicine, The University Hospital Rigshospitalet, Copenhagen, Denmark; Department of Pediatric and Adolescent Oncology, Villejuif, France; Dana-Farber/Boston Children’s Cancer and Blood Disorders Center, Boston, Massachusetts, USA; Department of Pediatrics, Harvard Medical School, Broad Institute, Cambridge, Massachusetts, USA; The Centre for Paediatric, Teenage and Young Adult Cancer, Institute of Cancer Sciences, University of Manchester, and Royal Manchester Children’s Hospital, Manchester, UK; Clinical Pediatric Oncology, Hopp Children’s Cancer Center (KiTZ), Heidelberg, Germany; Heidelberg University Hospital, Heidelberg, Germany; German Cancer Research Center, National Center for Tumor Diseases (NCT), Heidelberg, Germany; Department of Neurosurgery, The Preston Robert Tisch Brain Tumor Center. Pediatric Neuro-Oncology, Preuss Laboratory for Brain Tumor Research, Durham, North Carolina, USA; Department of Diagnostic Imaging, The Hospital for Sick Children, Toronto, Ontario, Canada; The Hospital for Sick Children and Department of Paediatrics, University of Toronto, Toronto, Ontario, Canada; UCL Great Ormond Street Institute of Child Health, Great Ormond Street Hospital for Children, London, UK

**Keywords:** child, low-grade glioma, MAPK, pediatric brain tumor, rebound, resistance, recurrence

## Abstract

Pediatric low-grade glioma (pLGG) is the most common childhood brain tumor group. The natural history, when curative resection is not possible, is one of a chronic disease with periods of tumor stability and episodes of tumor progression. While there is a high overall survival rate, many patients experience significant and potentially lifelong morbidities. The majority of pLGGs have an underlying activation of the RAS/MAPK pathway due to mutational events, leading to the use of molecularly targeted therapies in clinical trials, with recent regulatory approval for the combination of BRAF and MEK inhibition for BRAFV600E mutated pLGG. Despite encouraging activity, tumor regrowth can occur during therapy due to drug resistance, off treatment as tumor recurrence, or as reported in some patients as a rapid rebound growth within 3 months of discontinuing targeted therapy. Definitions of these patterns of regrowth have not been well described in pLGG. For this reason, the International Pediatric Low-Grade Glioma Coalition, a global group of physicians and scientists, formed the Resistance, Rebound, and Recurrence (R3) working group to study resistance, rebound, and recurrence. A modified Delphi approach was undertaken to produce consensus-based definitions and recommendations for regrowth patterns in pLGG with specific reference to targeted therapies.

**Key Points**

- Unresectable pediatric low-grade glioma (pLGG) is a chronic disease with periods of tumor stability and regrowth both on and off treatment.
- pLGGs are characterized by MAPK activation, prompting clinical trials targeting this pathway.
- pLGG regrowth may reflect resistance to treatment, recurrence off treatment, or rapid rebound growth within 3 months of discontinuing therapy.

Pediatric low-grade gliomas (pLGG) are the most common childhood central nervous system tumors and represent a major cause of lifelong morbidity for affected individuals in this age group.^1^ PLGGs have a variable natural history, similar to other chronic diseases, which is characterized by variable responses to treatment and periods of tumor stability punctuated by episodes of regrowth. In the chemotherapy era, although objective responses were seen, the major focus was on tumor progression, which can occur while on treatment because of “resistance” or as a “recurrence” after stopping treatment. Each of these regrowth states have its own etiologies, including the underlying molecular driver and interaction between the tumor and its host microenvironment.^2,3^

In the era of molecularly targeted therapies, specifically focused on the MAPK signaling pathway, dramatic responses and improved progression-free survival have led to the recent Food and Drug Administration approval of dabrafenib/trametinib combination therapy for pediatric patients 1 year of age and older with *BRAF*^V600E^-mutated pLGG requiring systemic therapy.^4,5^ Other trials have demonstrated promising activity of a variety of MAPK pathway-targeted inhibitors,^6–11^ with good patient tolerability. However, new challenges have emerged, as rapid progression has been observed following discontinuation of MAPK inhibition. In one retrospective series, 13/17 (76.5%) experienced rapid progression with a median time to progression of 2.3 months (range, 0.3–20.8 months).^12^ In this series, 90% of patients with pLGGs who progressed after BRAF inhibition was discontinued responded if rechallenged with BRAF inhibition alone or combined with MEK inhibition.^13,14^ A retrospective series from Boston reported rapid regrowth after cessation of therapy in patients with both BRAF fusions/duplications and *BRAF*^V600E^-mutant pLGGs treated with trametinib (2.38 and 2.86 months), respectively.^15^ This study reported that rapid growth, as measured by volumetric analysis, in some of the *BRAF*^V600E^-mutant LGG cohort peaked and then decreased without further therapy, which the authors proposed could represent a mixture of true progression and transient growth (pseudo-progression). The term “rebound” regrowth has started to be used following these reports without any definition or debate as to whether this represented a true different pattern of tumor growth after discontinuation of therapy distinct from that well known to occur in some pLGG patients after stopping conventional cytotoxic chemotherapy. It remains at present unclear as to if “rebound” regrowth is a distinct phenomenon seen with targeted therapies, whether this is a biological mechanism of action. Early preclinical data, from patient-derived cells, suggests that there is a difference between standard-of-care chemotherapy (vincristine and carboplatin) versus the *BRAFV600E*-specific inhibitor dabrafenib upon cellular proliferation and MAPK pathway reactivation upon withdrawal.^16^ But the data also suggest that growth rebound might not only be caused by a fast reactivation of the MAPK pathway but also by other mechanisms, eg, accumulation of upstream activators due to loss of negative feedback or parallel pathways.

These new tumor behaviors may be a result of different mechanisms that require specific clinical interventions. As such defining these may benefit the scientific and clinical communities. The terms “resistance,” “rebound,” and “recurrence” are actively used in the context of pLGG. However, these definitions have not been well established and the underlying biological mechanisms of each type of regrowth remain to be elucidated. In order to add these definitions to the progression and progression-free survival clinical endpoints, which use criteria such as those used by the Response Assessment in Pediatric Neuro-Oncology (RAPNO) working group,^17^ it is essential for the academic community try to establish consistent terminology for pLGG growth patterns that reliably and reproducibly inform clinical trials and the future optimal management.

Therefore, as part of the International Pediatric Low-Grade Glioma Coalition (iPLGGc) we formed the iPLGGc R^3^, “resistance, rebound, and recurrence” group and undertook a modified Delphi consensus approach which was undertaken to define such mechanisms. Delphi is a research survey technique using a consensus-based approach to deal with divergent opinions concerning real-world knowledge on a certain topic, employing experts within their domain of expertise.^18–20^

## Materials and Methods

As part of the iPLGGc R^3^ group, an initial virtual workshop was convened to discuss the 3 proposed patterns of pLGG tumor growth as follows:

### What is Resistance?

- Should this term only be used for those individuals who progress on treatment, or should it include those who do not respond initially or to rechallenge a given therapy?
- Is resistance defined by tumor progression or also by lack of objective response or stable disease eg, innate refractory or partial resistance?

### What is Recurrence?

- Is this term restricted to only patients who have entered a period of remission, or can it be used for pLGG in terms of regrowth after a period of tumor response or stability after treatment?
- Is there a typical time period and clinical pattern of tumor regrowth for pLGG?

### What is Rebound?

- How does this differ from “typical” pLGG tumor regrowth/ recurrence?
- Is this defined only by rapid progression after discontinuation of therapy?
- Is this specific to MAPK inhibitors or does this occur with other treatments?
- Does this need to be confirmed by response to rechallenge of the same drug or drug class?
- Should this be based on imaging evidence only and using what criteria?

The conclusion of the first iPLGGc R^3^ workshop was that there was not sufficient data and evidence to be able to define resistance, rebound, and recurrence and that a Delphi technique as a method of systematically collating expert consultation and building consensus was justified. This workshop formed the first stage of a modified Delphi process to define the problem and outline the questions to be addressed. Stages 2 and 3 consisted of 2 consensus-building survey rounds using a repeated iterative group survey process to try to achieve convergence of opinion. Finally, a fourth-stage consensus meeting was convened to understand any remaining divergence. The Delphi study was registered as a service evaluation at Great Ormond Street Hospital, London, the host organization of the senior author.

### Delphi Study Conduct

#### Selection of experts.—

**Figure 1.**
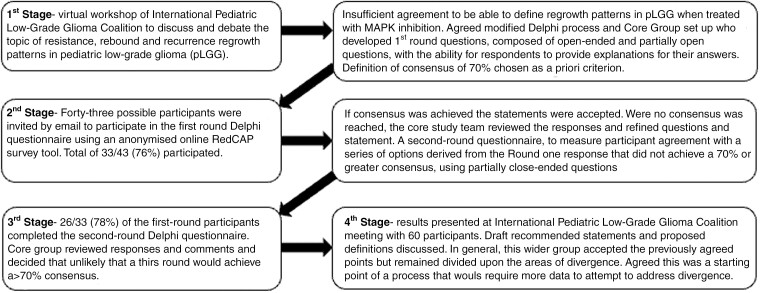
Summary of Modified Delphi Process

Minimum group size for a Delphi consensus has not been established, but we estimated that 20–30 experts in pediatric neuro-oncology would be an appropriate sample. Figure 1 Forty-three possible participants were invited by email to participate in the first-round Delphi questionnaire. These were selected as members of the iPLGGc R^3^ working group and additional members of the European SIOPe Brain Tumor Group representing pediatric neuro-oncology, neuropathology, and neuroradiology across Australia, Europe, Asia, and North America. Positive responses to this email were included in the second Delphi questionnaires. A subset of these positive responders participated in the second iPLGGc R^3^ workshop.

#### Informational input.—

Following the first stage workshop, a first-round questionnaire was designed, prefaced by a briefing document informed by a comprehensive literature review by the core study team (DH, UT, TC, and POH). Questions and statements were illustrated by a clinical vignette.

#### Definition of consensus and avoidance of bias.—

Of 70% was chosen as a priori criterion for consensus. Email invitations were sent with a URL hyperlink allowing anonymous electronic completion using a RedCAP survey tool. The deadline for completing the questionnaires was set at 5.5 weeks after sending the hyperlink. Non-responders were sent a general reminder every Monday until the week of the deadline. Initial questionnaire was composed of open-ended and partially open questions, with the ability for respondents to provide explanations for their answers. If consensus was achieved the statements were accepted. Were no consensus was reached, the core study team reviewed the responses and refined questions and statements for a second-round questionnaire. The purpose of the second questionnaire was to measure participant agreement with each of the options derived from the Round one response that did not achieve a 70% or greater consensus. The second questionnaire was made up of partially close-ended questions which requested participants revisit each vignette, consider the different options and then select the options that they would choose if faced with that vignette. After the second round the core study team again reviewed responses and it was decided that a third round was unlikely to resolve the remaining questions and to move to the fourth stage consensus meeting to better understand any remaining divergence.

#### Consensus meeting.—

The fourth and final stages were in-person consensus meetings, facilitated by the core study team, presenting the experts with outcomes from the first and second questionnaires, to explore and understand why options were selected and deepen understanding of reasoning behind variations.

#### Data analysis.—

A combination of qualitative and quantitative methods was used in analysis of the completed questionnaires from each round. Semi-structured content analysis has been used across all rounds to summarize open-ended questions or other written responses.

## Results

In the first stage initial R^3^ iPLGGc workshop, it was agreed that these guidelines would focus on targeted approaches and the development of a modified Delphi study to seek expert consensus on the definition of patterns of pLGG regrowth while on those therapies. Based on discussions in the workshop the core study team initially devised proposal descriptions for resistance, rebound, and recurrence regrowth as shown in Table 1, and this was shared with the group.

**Table 1.** Proposed Definitions/ Statements Following Initial R^3^ Workshop

Pattern of regrowth	Proposed Definitions/ Statements following initial R^3^ workshop
Resistance	Proposal to define resistance as growth while on treatment (ie, MAPK inhibitor therapy), using RAPNO pLGG criteria (≥25% increase in size) ideally confirmed with a second scan if clinically appropriate.^17^ Stable disease is considered resistance.
Rebound	This is a new concept and more complex to define, but proposed rebound is a more rapid growth of an existing lesion within 6 months of stopping therapy using RAPNO pLGG criteria (≥25% increase in size).^17^
Recurrence/Regrowth	Many pLGG will have periods of stability after treatment stopping followed by possible regrowth over a wide period. To differentiate recurrence regrowth from resistance and rebound propose to define it as growth (≥25% increase in size using RAPNO pLGG criteria) or a new lesion that appears more than 6 months after therapy is discontinued.^17^ Tumors that grow rapidly may benefit from biopsy, as rapid growth in recurrence is atypical of pLGG.

Using these proposed definitions and statements the core study team devised questions for the second stage and first Delphi survey round. The questions for the Delphi rounds 1 and 2 as administered anonymously using a RedCAP survey tool are detailed in Supplementary Data.

A total of 33 of the 43 (76%) invited experts participated in the first questionnaire round, comprising 16 pediatric oncologists, 12 pediatric neuro-oncologists, 3 neuro-radiologists, and 2 pediatric neurologists. Two respondents did not answer all the questions and so for some questions, there are only 31 responses.

A total of 26 of the 33 (78%) invited experts participated in the third stage, second questionnaire round, including 14 pediatric oncologists, 9 pediatric neuro-oncologists, 2 pediatric neuro-radiologists, and 2 pediatric neurologists. One respondent did not answer all the questions.

Table 2 summarizes the Delphi Survey responses for both rounds 1 and 2. Six questions did not achieve a 70% or greater consensus and the core study team reviewed the anonymous comments and explanations for votes and revised these 6 non-consensus questions in a second round. Three questions achieved greater agreement and therefore consensus. However, for 3 outstanding questions, although a greater percentage of respondents agreed, the 3 questions still failed to pass the 70% threshold. There were no obvious differences in discrepancy of opinions as per respondents’ discipline but the numbers were too small to statistical analyze on a specialty basis.

**Table 2.** Summary of Delphi Round 1 and 2 Survey Responses

Statements where consensus was achieved.				
Consensus statement	Round 1 Delphi		Round 2 Delphi	
	*N*	% agreement	*N*	% agreement
*General statement*				
There is benefit in applying universal definitions of growth across pLGG molecular subtypes (ie, NF1/BRAFV600E/BRAF fusion, etc.)	22/31	**71**		
Universal definitions in growth should be applied across all RAS/MAPK inhibitors	27/31	**87**		
When interpreting growth, timing, scan sequences, and slice thickness should follow pLGG RAPNO criteria.	29/31	**93.5**		
*Resistance*				
The scan which demonstrates the best recorded MRI response while on MAPK inhibitor therapy is considered the best can for comparison	17/33	52	19/26	**73**
Resistance is defined as ≥25% tumor growth while on MAPK inhibitor therapy. (as per pLGG RAPNO criteria ie, product of biperpendicular diameters)	33/33	**100**		
Development of new metastatic disease while continuing MAPK inhibitor therapy is defined as resistance	28/33	**85**		
*Rebound*				
The last MRI scan while on MAPK inhibitor therapy should be the scan which is used to calculate percentage growth in tumor after stopping MAPK inhibitor therapy	26/31	**84**		
Rapid growth (rebound) is defined as ≥ 25% growth in tumor in 3 months following cessation of MAPK inhibitor therapy	29/31	**88**		
Development of new metastatic disease after stopping MAPK inhibitor therapy is NOT considered rebound growth, but is rather considered progression	18/31	52	21/26	**80**
In the definition of rebound growth, if a patient is rechallenged with MAPK inhibitor therapy after rebound growth, they should demonstrate response within 6 months of rechallenging with MAPK inhibitor therapy.	14/31	45	25/25	**100**
*Regrowth/recurrence*				
The last MRI while on MAPK therapy should be the scan which is used to calculate percentage growth in the tumor after stopping MAPK inhibitor therapy.	24/31	**77.4**		
Recurrence regrowth should be mutually exclusive from rebound growth	27/31	**87.1**		
If the tumor grows more than 6 months after cessation of MAPK inhibitor therapy, this should be considered to be classical recurrence/ progression and NOT rebound growth.	22/31	**71**		

Statements where consensus is not reached.				
Statement	Round 1 Delphi		Round 2 Delphi	
	N	% agreement	N	% agreement
When considering an optimal timeframe to perform the first scan after discontinuing MAPK therapy, do you perform the next scan more than 8 weeks and less than or equal to 12 weeks after stopping MAPK inhibitor therapy. *(The other option was to perform the next scan less than 8 weeks after stopping MAPK inhibitor therapy.)*	18/31	58	17/25	68
In order to differentiate between a tumor that has acquired resistance to MAPK inhibitor therapy versus rebound growth, but remains responsive to MAPK inhibitors, it is beneficial to rechallenge the patient with MAPK inhibitor to confirm rebound growth.	14/31	45	15/25	60
There is utility in providing consensus radiographic-only definitions of pLGG growth as a first step while in parallel establishing clinical/ functional definitions of progression?	20/31	65	16/25	64

Bold represent consensus achieved where % agreement was equivalent of greater than 70%.

The fourth and final stage was a consensus meeting which was held at the most recent iPLGGc meeting of the entire group in Atlanta, USA, in November 2022. There were more than 60 people in attendance including both Delphi and non-Delphi participants and the results from the Delphi rounds 1 and 2 were presented by the core study team.

In general, this wider group accepted the previously agreed points but remained divided upon the areas of divergence. In particular, the question of when to rescan after stopping treatment with a MAPK inhibitor did not achieve agreement on a specific time frame. Some believed this may be influenced by individual factors, including (1) mutation type as *BRAFV*^*600E*^ mutant pLGGs may have different regrowth kinetics than fusion-BRAF tumors, (2) location of tumor within a vital area where risk of irreversible clinical damage can occur, and (3) whether the patient is on a clinical trial. Therefore, a pragmatic proposal was put forward to plan to scan at least 3 months as per standard practice but sooner based on clinical concerns, since rebound growth has been reported to occur earlier following discontinuation of MAPK inhibitors. The issue of requiring rechallenge with a MAPK inhibitor and demonstrating a response to confirm rebound and exclude resistance was strongly debated, but no agreement was achieved. Some argued that patients may have an initial rebound on scan, but this may stabilize, and patients may not require rechallenge. Others may wish to change the specific MAPK inhibitor if rebound was significant, and this would complicate interpretation. It was decided not to include rechallenge in the definition of rebound and instead focus on regrowth patterns. However, it was generally agreed that current imaging using for example RAPNO pLGG criteria is an objective way of measuring regrowth and hence the definitions proposed are based on imaging. It was strongly emphasized that clinical and functional endpoints are critical for a chronic disease such as pLGG. For example, in patients with optic pathway glioma, a deterioration in visual function may occur with or without imaging changes but may still lead one to consider a change in therapy. Pragmatically it was agreed that the imaging-based criteria would be proposed but when feasible objective clinical and functional endpoints should be considered and included.

It was noted that regrowth between 3 and 6 months following the discontinuation of MAPK inhibitor therapy could not be confidently determined to be rebound or recurrence. More data is needed to answer this gap in knowledge. In addition, the issue of how to consider the cystic components of the tumor as part of the definition of progressive disease (PD) was discussed and participants agreed to follow the detailed criteria used in the RAPNO pLGG criteria.^17^

## Recommendations

The working consensus-based definitions for resistance, rebound, and recurrence for PLGG treated with MAPK inhibitors arising from this modified Delphi approach are reported in Table 3.

**Table 3.** Consensus-Based Definitions for Resistance, Rebound, and Recurrence Regrowth Patterns in Pediatric Low-Grade Glioma: International Pediatric Low-Grade Glioma Coalition

Consensus statements and definitions	Resistance	Rebound	Recurrence regrowth
Definitions and statements apply to all pediatric low-grade glioma molecular and pathology subtypes. (ie, NF1/*BRAF*^*V600E*^/fusion BRAF, pilocytic astrocytomas, gangliogliomas, etc.)			
Definitions and statements apply to all RAS/MAPK inhibitors.			
Recommend scan sequences and slice thickness plus interpretation as per RAPNO pediatric low-grade glioma criteria (RAPNO pLGG) Note if tumor has cystic and solid components follow the detailed guidance on cystic disease as recommended by RAPNO pLGG.^17^			
Timing in relation to treatment	Resistance is growth while *on systemic treatment* eg, MAPK inhibitor therapy.	Rebound growth of an existing lesion *usually within 3 months* of cessation of systemic (MAPK inhibitor) therapy. Regrowth ≥ *6 months* after stopping treatment is *NOT rebound*.	Recurrence regrowth occurs *off treatment* and is the term to be used for any regrowth ≥ *6 months* after stopping treatment.
Radiological criteria as per RAPNO pLGG. (Cross-sectional change)	*≥25% of growth or a new (metastatic) lesion*, ideally confirmed with a second scan, unless clinically inappropriate. Stable disease should NOT be called resistance.	*≥25% of growth of an existing lesion*, ideally confirmed with a second scan unless clinically inappropriate.	*≥25% of growth or a new (metastatic) lesion*, ideally confirmed with a second scan, unless clinically inappropriate.
MRI scan to be used for comparison. (To calculate percentage regrowth)	Best recorded MRI response while on MAPK inhibitor therapy.	The last MRI scan was while on MAPK inhibitor therapy.	The last MRI scan was while on MAPK inhibitor therapy.
New (Metastatic) lesion	A new (metastatic) lesion occurring on treatment is regarded as resistance regrowth.	New metastatic lesions are NOT considered rebound regrowth.	A new (metastatic) lesion occurring off treatment is regarded as recurrence regrowth.

## Discussion

Pediatric low-grade gliomas are a heterogeneous group of brain tumors characterized by activation of the MAPK pathway. Molecular-based targeted therapies are increasingly being studied and starting to be approved for some pLGG subsets, but there remain many questions to be answered including optimal duration, uncertainty about long-term toxicity as a result of prolonged exposure to these therapies potential to develop acquired resistance and the mechanism involved. While the focus of the discussion was on MAPK inhibitors (ie, MEK, BRAF, and RAF inhibitors), the group acknowledged some pLGG harbor mutations in *NTRK* and *FGFR* genes and similar patterns of regrowth may be encountered with specific targeted inhibitors of these pathways and this needs to be considered as more data emerges from clinical trials and experience of these novel targeted drugs. Therefore, there is a pressing need to redefine the responses of pLGG to those therapies to enable future mechanistic research, clinical trial design, and precision-based approaches to the management of these tumors.

The most debated regrowth pattern was the idea of “rebound” regrowth. The iPLGGc debated the difference between rebound and recurrence, how to define rebound, and whether rebound was only seen following targeted MAPK inhibitor therapies or whether this was seen with other existing treatments such as cytotoxic chemotherapy or Bevacizumab. At the first stage of the Delphi process, it was proposed that rebound was rapid growth (≥25%) of an existing lesion following therapy cessation, noted most often in the context of MAPK inhibitor discontinuation and had been reported as early as 2 weeks and usually within 3 months. The full Delphi process further refined this definition to exclude new (metastatic) lesions which would be considered recurrence. The last MRI scan after stopping treatment (MAPK inhibitor therapy) was preferred as the best scan to assess percentage regrowth based on RAPNO pLGG criteria.^17^ Although it has been noted that patients with rebound regrowth nearly always will respond to rechallenge with the same or similar MAPK inhibitor therapy, no consensus was reached on whether such rechallenge response was required to be included to define rebound. The inclusion of clinical functional parameters was recognized to be important in pLGG, but only radiological criteria have been applied at this time. The specific issue of how to deal with solid/ cystic progression is complex in pLGG and the group decided that the guidance in RAPNO pLGG should be used.^17^ Finally, the iPLLGc suggests that rebound occurs within 3 months of discontinuing therapy and recurrence occurs more than 6 months following therapy. However, regrowth 3–6 months following therapy is not clearly defined.

With respect to tumor recurrence, it should be appreciated that the impact of discontinuing tyrosine kinase inhibitors (TKI) in patients who have achieved a complete remission or who have no evaluable disease has been studied in adults with gastrointestinal stromal tumors, renal cell carcinoma, and advanced sarcomas with a high rate of tumor recurrence, but not 100%, a high rate of response upon rechallenge and in some cases with a reported rapid and increased growth rate post discontinuation.^21–24^ Rapid tumor flare also known as the flare phenomenon has been reported in renal cell carcinoma patients with brain metastases upon dose interruption of sunitinib during the 2-week off-treatment period of the standard sunitinib dosing schedule,^22,23,25^ in EGFR-mutant lung cancer patients who discontinued erlotinib or gefitinib due to acquired resistance (23% flare rate at a median of 8 days, range 3–21 days during the EGFR TKI washout period)^26^ and in thyroid cancer after withdrawal of both sorafenib and lenvatinib.^27–29^ In some cases, this is thought to be related to rapid revascularization and or tumor edema after reversal of VEGF inhibition with preclinical and clinical data supporting this mechanism. As per RAPNO pLGG, potential revascularization is one reason not to rely on contrast enhancement^17^ but T2/ FLAIR sequences and may be a reason some have reported that “rebound” could be a temporary effect seen shortly after stopping treatment^15^ and that if clinically well a second confirmatory MRI scan may be appropriate to confirm ongoing regrowth before restarting any further therapy.

In terms of the risk of recurrence and possible rebound regrowth after discontinuing MAPK inhibition prior to PD, there is limited data, as TKIs are usually continued indefinitely until PD or other treatment-limiting event. In the *BRAF*^V600E^ mutated melanoma COMBI-d and COMBI-v clinical trials patients who received dabrafenib plus trametinib, 23% of responders discontinued drug prior to PD, and were observed to have a median time to PD of 3.7 months from cessation.^30^ Other retrospective studies report recurrence rates prior to PD that vary widely from 0% to 69% with a median time from cessation to progression between 2.5 and 22 months but these results are hampered by delayed detection in asymptomatic patients related to scanning intervals and small cohort sizes.^31–37^ It has also been suggested that initial treatment duration prior to TKI discontinuation may have an impact on regrowth with a trend for improved progression-free survival after cessation in patients with longer treatment duration but no prognostic factors consistently identified.^31^ There remains a need for a predictive marker to identify patients who can safely cease targeted therapy in melanoma, with plasma cell-free tumor DNA (cfDNA) offering some promise in melanoma.^38^ Similar methods now being investigated in melanoma and papillary craniopharyngioma may eventually be used for pLGG to guide treatment duration.^39,40^ However, concerns remain about the sensitivity of blood-based analysis for primary brain tumors, and liquid biopsy of cerebrospinal fluid (CSF) may offer increased sensitivity at the cost of a more invasive procedure.^41,42^

It must be emphasized that this is a Delphi approach and although we attempted to follow guidance on the conduct of this methodology the results are a consensus of invited but essential self-selected experts. As such the recommendations arising are more experienced than evidence-based and represent a starting point rather than definitive guidelines, which may help to provide a framework for future data collection.

There may be other patterns of tumor regrowth that the R^3^ have not yet considered. Both clinical trials and real-world reports show that “treatment beyond progression” with MAPK inhibitors has proven in some cases to be successful with prolonged patient benefit.^43^ For some time, oncologists have raised the possibility that PD as defined by established response criteria may not mean treatment failure,^44^ with the best-established example being immunotherapy and the subsequent development of specific criteria such as iRECIST and iRANO.^45–47^ Possible “pseudoprogression (PsP)” is now recognized in both adult and pediatric low-grade glioma post-radiation therapy for both photon-based and proton beam therapy.^48,49^ It is possible that a type of PsP may occur in pLGG patients being treated with MAPK inhibition, related to tumor response with cell death promoting inflammatory signals resulting in edema and cellular infiltrates that can be seen as a transitory increase in tumor size on imaging which subsequently resolves. This may occur early in treatment or maybe an explanation for later isolated PD, often in a small residual following a previous significant objective response. This can lead to premature cessation of an effective therapy. In our proposals, we have suggested a second confirmatory MRI scan if clinically appropriate prior to stopping treatment to help confirm regrowth patterns. The possibility of PsP as a regrowth pattern outside of radiotherapy in pLGG needs further evaluation.

## Conclusions

The advent of targeted therapy for pLGG while providing the possibility of more effective and kinder therapy for patients, also raises many questions. Here we present a modified Delphi approach to build international consensus-based definitions for resistance, rebound, and recurrence regrowth patterns in pediatric low-grade glioma treated by MAPK inhibition. We emphasize this is a starting point as it was not possible to achieve consensus for all items and reflects only the views of a select group of experts based on current available data and experience. It is clear this is a process and will need to be revisited with more data and wider participation in the future but represents and initial attempt to raise awareness and provide some initial recommendations. Other questions include the optimal duration of treatment, when to discontinue therapy and when to rechallenge. There are also theoretical concerns about the possible impact of MAPK inhibitors on the natural history of pLGG senescence, where the tendency is for natural tumor growth arrest with increasing age. In addition, the financial impact on individuals and healthcare systems, which can be positive and negative, must be considered and studied holistically.

We hope that the iPLGGc R^3^, along with the preclinical modeling, clinical trials, and quality of life/late effects working groups and the wider academic community will continue to provide insight and answers to these developing questions.

## Supplementary material

Supplementary material is available online at *Neuro-Oncology* (https://academic.oup.com/neuro-oncology).

noae074_suppl_Supplementary
