# Supplementary material for: Resistance, rebound, and recurrence regrowth patterns in pediatric low-grade glioma treated by MAPK inhibition: A modified Delphi approach to build international consensus-based definitions—International Pediatric Low-Grade Glioma Coalition
Source: Neuro Oncol. 2024 May 14;26(8):1357–66. doi: 10.1093/neuonc/noae074 (PMC11300023; doi:10.1093/neuonc/noae074)
Supplement: noae074_suppl_Supplementary [file noae074_suppl_supplementary.docx]

Supplementary information

DELPHI QUESTIONNAIRE ROUND 1

**Introduction**

Thank you for agreeing to complete the Pediatric Low-Grade Glioma Tumor Regrowth Questionnaire.

The objective of this questionnaire is to understand how physicians approach the understanding of pediatric low-grade glioma growth on and/or after MAPK inhibitor therapy. In order to allow for biological insights as well as uniform multi-institutional clinical study methodology, we suggest the definition of 3 growth patterns that represent mechanistic differences: resistance, rebound, and recurrence regrowth/natural. The Pediatric Low-Grade Glioma Resistance/Rebound and recurrence “R^3^” Subcommittee consisting of 35 pediatric neuro-oncologists, scientists, neuroradiologists met iteratively to develop working definitions, using collective experience and a comprehensive review of the literature. The workshop was beneficial in proposing definitions of each growth pattern, however it did not achieve a complete consensus. A Delphi approach has therefore now been adopted to further achieve consensus on definitions. A summary of the workshop consensus definitions are provided prior to each section. Your response to all questions should reflect how YOU would define the growth, which might or might not be consistent with the Subcommittee’s, your colleagues or current published guidelines. *Please use* *the comment box after each question to briefly describe the rational for your clinical* *decisions. You may choose to save your responses and work on the survey again at a* *later date.*

**Part 1 – Resistance**

The Subcommittee proposes to define resistance as growth while on treatment i.e., MAPK inhibitor therapy. To use current radiological (RAPNO LGG criteria) of >25% of growth or a new lesion, ideally confirmed with a 2^nd^ scan unless clinically inappropriate. Stable disease should not be called resistance.

1. How would you define tumor resistance while on MAPK therapy? Tick all that apply.
   1. >25% tumor growth
   2. Development of new metastatic disease
   3. <25% growth

Comments: __________

1. What comparison scan would you use to calculate percent growth while on MAPK therapy?
   1. Last MRI prior to treatment initiation
   2. Best recorded MRI response

Comments: __________

1. Do you agree that stable disease (<25% change) during therapy is not considered resistance?
   1. Yes
   2. No

Comments: _____________

**Part 2 – Rebound**

The Subcommittee appreciates that rebound growth is a new concept and more complex to define. We propose that rebound is a more rapid growth of at least 25% of an existing lesion from therapy cessation in less than 6 months of therapy. To use current radiological (RAPNO LGG criteria) of >25% of growth.

1. Within what length of time from cessation of MAPK therapy would growth need to occur for you to consider it rebound growth?
   1. < 3 months
   2. < 6 months (more than one assessment)
   3. < 12 months

Comments: __________

1. How would you define rapid growth immediately following cessation of MAPK therapy? Circle all that apply.
   1. >25% tumor growth in 3 months
   2. >50% tumor growth in 3 months
   3. >25% tumor growth in 6 months
   4. >50% tumor growth in 6 months
   5. <25% tumor growth in 3 months

Comments: _________

1. Is new metastatic disease after stopping therapy considered rebound?
   1. Yes
   2. No

Comments: ____

1. What scan would you use to calculate percent growth after stopping MAPK therapy?

- 1. Last MRI while on MAPK therapy
  2. First MRI after discontinuation of MAPK therapy
  3. Best recorded MRI response

Comments: __________

1. How soon after stopping MAPK therapy should you perform the next scan.
   1. Less than or equal to 6 weeks
   2. More than 6, less than or equal to 8 weeks
   3. More than 8, less than or equal to 12 weeks
   4. Greater than 12 weeks

Comments: ____________

1. Do you feel stabilization/response post rechallenge with MAPK therapy is necessary to define rebound growth?
   1. Yes
   2. No

Comments: ___________

1. If rechallenged with MAPK therapy, how soon does a tumor need to show response/stabilization to define rebound growth?
   1. < 3 months
   2. < 6 months

Comments: ___________

**Part 3 – Recurrence regrowth**

Many pLGG will have periods of stability after treatment stopping followed by possible regrowth over a wide period. To differentiate recurrence regrowth from resistance and rebound the Subcommittee propose to define it as at least 25% of growth or a new lesion, ideally confirmed in at least 2 scans at 6 months or great post therapy discontinuation. NB For tumors with very large regrowth e.g., double the size of the previous scan consider a biopsy prior to restarting therapy initiation as this raises concern tumor is not typical of the natural history of PLGG.

1. Do you agree regrowth/natural progression should be mutually exclusive from rebound growth?
   1. Yes
   2. No

Comments: ________

1. How far from MAPK cessation would growth need to occur for you to consider it delayed natural progression and not rebound growth?
   1. > 3 months
   2. > 6 months
   3. > 12 months

Comments: ________

**Part 4: General**

1. Do you agree in applying universal definitions of growth across molecular subtypes?
   1. Yes
   2. No

Comments: _________

1. Do you agree in applying universal definitions in growth across RAS/MAPK inhibitors?
   1. Yes
   2. No

Comments: _____

1. Do you agree, there is utility in providing working radiographic only definitions of pLGG growth?
   1. Yes
   2. No

Comments: ________

1. Do you agree when interpreting growth that timing, scan sequences, and splice thickness should follow pLGG RAPNO criteria?
   1. Yes
   2. No

Comments: _________

DELPHI QUESTIONNAIRE ROUND 2.

**Question1**

In the previous Delphi survey, we asked: *“When calculating percentage growth of a tumor while on MAPK therapy, which scan would you use as your baseline for comparison?”*

The first-round responses are listed below:

a. Last MRI prior to treatment initiation (16/33: 48%)

b. Best recorded MRI response (17/33: 52%)

A consensus was not reached and therefore these questions need to be asked again. Those who chose a nadir of best recorded response (52%) felt that in scenarios where a large shrinkage of tumor initially occurred leaving a small residual, waiting on the percent growth from the last MRI prior to treatment may cause delays in detection of resistance. They cited similar practice to procedures in RANO/RAPNO assessment of response. Those who chose last MRI prior to treatment (48%) felt that in scenarios where a large shrinkage of tumor initially occurred leaving small residual, the percent growth from the best recorded response may overestimate meaningful growth. If the tumor size remained less than the baseline at start of treatment, then the clinician would feel comfortable continuing treatment.

**REVISED QUESTION 1**

Reflecting on the responses of the group, would you agree to provide a consensus agreement on calculating percentage growth of a tumor while on MAPK treatment; that the best scan for comparison should be:

- - 1. Best recorded MRI response while on MAPK inhibitor therapy.
    2. Last MRI prior to treatment initiation with MAPK inhibitor therapy

**Question 2**

In the previous Delphi survey, we asked: *“Is new metastatic disease after stopping therapy considered rebound?”*

The first-round responses are listed below:

- 18/31: 58% said No.
- 13/31: 42% said Yes.

A consensus was not reached and therefore this question needs to be asked again. The majority of respondents (58%) felt that new metastatic disease developing after stopping MAPKi treatment was not considered rebound. They cited that any new disease not present during prior MAPKi treatment should be considered progression. Rebound would therefore be limited to a predefined target lesion present during previous MAPKi treatment. For those respondents who considered new disease as rebound, they cited that it may be because a small undetected lesion had been kept quiescent during MAPKi treatment and only became apparent on release of tumor from MAPKi inhibition.

**REVISED QUESTION 2**

In considering the definition of rebound growth, do you agree that development of new metastatic disease after finishing MAPK inhibitor therapy, is NOT considered rebound growth, but is rather considered progression.

1. Yes
2. No

**Question 3**

In the previous Delphi survey, we asked: *“How soon after stopping MAPK inhibitor therapy, should you perform the next scan?*”

The first-round responses are listed below:

1. Less than or equal to 6 weeks. (8/31: 26%)
2. More than 6, less than or equal to 8 weeks. (5/33: 16%)
3. More than 8, less than or equal to 12 weeks (18/33. 58%)
4. Greater than 12 weeks (0/31: 0%)

A consensus was not reached and therefore we need to ask the question again. The majority of respondents (58%) chose 8-12 weeks as the preferable timeframe to perform the next scan after stopping MAPK therapy. The rationale included having a “less is more” approach in the context of pLGG and an intention to scan close to, but less than 3 months from the end of treatment. Additional comments related to some difference in behavior which may be related to molecular profile of the tumor, for example those with BRAFV600E mutations may want to scan these patients earlier. For those respondents that picked earlier time frames (42% said less than 8 weeks), they quoted personal experience of patients showing rapid growth within weeks of discontinuation of MAPK therapy.

**REVISED QUESTION 3**

When considering an optimal timeframe to perform the first scan after discontinuing MAPK therapy, should you perform the next scan:

1. Less than 8 weeks after stopping MAPK inhibitor therapy.
2. More than 8 weeks, less than or equal to 12 weeks after stopping MAPK inhibitor therapy.
